# HDCTfusion: Hybrid Dual-Branch Network Based on CNN and Transformer for Infrared and Visible Image Fusion

**DOI:** 10.3390/s24237729

**Published:** 2024-12-03

**Authors:** Wenqing Wang, Lingzhou Li, Yifei Yang, Han Liu, Runyuan Guo

**Affiliations:** 1 School of Automation and Information Engineering, Xi’an University of Technology, Xi’an 710048, China; 2 Shaanxi Key Laboratory of Complex System Control and Intelligent Information Processing, Xi’an University of Technology, Xi’an 710048, China

**Keywords:** image fusion, infrared image, visible image, Transformer, CNN

## Abstract

The purpose of infrared and visible image fusion is to combine the advantages of both and generate a fused image that contains target information and has rich details and contrast. However, existing fusion algorithms often overlook the importance of incorporating both local and global feature extraction, leading to missing key information in the fused image. To address these challenges, this paper proposes a dual-branch fusion network combining convolutional neural network (CNN) and Transformer, which enhances the feature extraction capability and motivates the fused image to contain more information. Firstly, a local feature extraction module with CNN as the core is constructed. Specifically, the residual gradient module is used to enhance the ability of the network to extract texture information. Also, jump links and coordinate attention are used in order to relate shallow features to deeper ones. In addition, a global feature extraction module based on Transformer is constructed. Through the powerful ability of Transformer, the global context information of the image can be captured and the global features are fully extracted. The effectiveness of the proposed method in this paper is verified on different experimental datasets, and it is better than most of the current advanced fusion algorithms.

## 1. Introduction

Due to the limitations of single-modality images, it is impossible to capture both the salient features and rich texture details of the target at the same time. Therefore, image fusion technology has received extensive attention and development [1,2,3]. Fused images of infrared and visible images have a wide range of applications in many fields, such as military reconnaissance, nighttime driving assistance, target detection, and semantic segmentation. Infrared images mainly reveal the thermal properties of the target object, while visible images contain rich texture structure and color information. Therefore, in certain application scenarios, the fusion of these two images can yield a composite image that encompasses both target information and intricate texture details.

Traditional fusion techniques, such as multi-scale transform-based methods [4,5], sparse representation-based methods [6,7], saliency-based methods [8], subspace-based methods [9], and hybrid methods [10], use different information processing methods to fuse images. However, traditional fusion methods suffer from information loss, are sensitive to scene changes, and have difficulty processing complex textures, which limits their performance in terms of fusion quality and scene adaptability. In contrast, among deep-learning-based approaches, convolutional neural network (CNN) [11], generative adversarial network (GAN) [12], and encoding–decoding architecture (AE) [13] are the main frameworks, which have the advantages of automatic learning features, flexibility, and robustness, and can more effectively capture image information and produce robust fusion results.

Although deep-learning-based fusion methods can generate excellent fused images, they also encounter certain limitations. Most of the fusion methods ignore inter-channel relationships and position information, and are unable to acquire both shallow and deep features, which results in the fused image not containing more information. In order to solve the above problems, the paper proposes a dual-branch infrared and visible image fusion network combining CNN and Transformer. The major contributions of this paper are as follows:

(1) This paper proposes a dual-branch network by combining CNN and Transformer, which is capable of simultaneously extracting local and global features, enhancing contextual information, and ensuring that the fused image contains richer contents.
(2) The proposed method constructs a CNN-based local feature information extraction module (LFEM). The ability of the network to extract texture details is enhanced using the residual gradient module. Coordinate Attention (CA) and jump links are also used, effectively linking shallow and deep features.
(3) The proposed method constructs a Global Feature Information Extraction Module (GFEM), which consists of adaptive Transfomers and convolutional layers. Since Transfomer is able to capture the global contextual information, it is able to fully extract the global feature information, which leads to perserve more features in the fused image.

## 2. Related Works

In this section, some existing image fusion algorithms are summarized.

### 2.1. Traditional Image Fusion Methods

Over the past many years, many traditional methods for fusing of infrared and visible images have been widely used, such as multi-scale transform-based methods [4,14], sparse representation-based methods [6,15], saliency-based methods [8,16], subspace-based methods [9], and hybrid methods [10,17], which process and fuse the images from different perspectives. These methods aim at fusing multiple images into a new image to obtain better visualization and extract richer information.

The methods based on multi-scale decomposition are to first use the same multi-scale decomposition methods to decompose the source image separately. Then, the decomposed low-frequency components and high-frequency components are fused using fusion rules. Finally, the fused components are inversely transformed accordingly to reconstruct the fused image [14].

The sparse-representation-based image fusion methods are founded on sparse representation theory. Utilizing the learned sparse dictionary, these methods represent multiple source images as sparse linear combinations on the dictionary. Subsequently, the fused image is generated through a weighted combination of these sparse representations [15].

The saliency-based methods are typically categorized into two types: weight calculation and saliency target extraction. The former usually involves combining multi-scale transformations to decompose the source image into detail and base layers. Subsequently, a saliency map is derived by applying a saliency extraction model to these layers. Next, the weighting map is obtained based on the saliency map to generate the fused base and detail images. Finally, the fused image is constructed based on these fused base and detail images. The latter method utilizes saliency detection to extract important area information from the source image. This extracted key information is then integrated into the final fused image [16].

The subspace-based image fusion method first decomposes the source images into bases and coefficients, then selects appropriate subspaces, merges the bases and coefficients, and finally reconstructs the fused image. This method effectively integrates the structural information and texture details of different source images to generate high-quality fused images [9].

The hybrid methods primarily leverage the strengths of various techniques and employ hybrid models to attain enhanced fusion outcomes. These methods usually combine different fusion technologies and can flexibly select the most suitable fusion strategy in different situations, thus producing more practical and robust fusion images [17].

The traditional infrared and visible image fusion methods have their own advantages and disadvantages. The multi-scale transformation-based methods can retain multi-resolution information and have flexible fusion rules. However, the algorithm performance overly relies on decomposition methods and fusion rules, resulting in poor adaptability. The sparse representation-based methods have theoretical advantages and better adaptability, but the dictionary is difficult to learn and the computation cost is high. The saliency-based methods can highlight the key information and combine it with the advantages of multi-scalability. The subspace-based methods can effectively integrate structure and texture. However, there are difficulties in subspace decomposition and selection. The hybrid methods can synthesize the advantages of various techniques and improve practicality, but the model design is complicated and the parameter adjustment is difficult. It is worth noting that these traditional methods often require manual setting of parameters and formulation of fusion rules, and only extract the shallow features. They may not be effective in dealing with complex scenes and do not yield optimal results.

### 2.2. Deep-Learning-Based Fusion Methods

Traditional image fusion methods rely on hand-designed feature extractors and simple models, have high requirements on data quality and features, and have limited generalization capabilities. In contrast, deep learning fusion methods can automatically learn higher-level feature representations from data through end-to-end learning, and have higher flexibility and generalization capabilities. Deep learning fusion methods can usually achieve better performance in different types of data and tasks. Deep-learning-based methods can be categorized into three groups: methods based on AE, methods based on CNN, and methods based on GAN.

The main idea of the AE-based method is to use a large number of image datasets to train an encoder and decoder, where the encoder is used for feature extraction from the image and the decoder is used for image reconstruction. Then, the designed fusion rules are used to fuse the extracted features, and finally, the fused image is reconstructed by the decoder. The DenseFuse fusion method is the classical AE-based method [18], which has a encoding network combined with convolutional, fusion, and dense block layers. The extracted features are fused using a fusion strategy. Finally, the image is reconstructed by the decoder. Li et al. proposed a fusion network based on residual structure, named as RFN-nest, using feature enhancement loss and detail preservation loss to guide training to improve fusion performance [19]. Han et al. proposed a learnable fusion rule based on pixel categorization saliency, which overcame the deficiency of manually formulated fusion rules and further improved the applicability of the network [20].

The fusion method based on CNN learns image features through tne convolutional neural network, and then uses fusion strategy to fuse multiple source images into the final image. With the learning ability of CNN network, it can automatically extract important features in the image and achieve high-quality image fusion. Ma et al. proposed an image fusion method based on saliency target detection named as STDFusionNet, which introduced a saliency target mask into the loss function to guide the training of the fusion network [21]. Tang et al. proposed an infrared and visible image fusion method for illuminating dark areas named as PIAFusion, which reasonably improved the fusion quality of nighttime images by removing the light brightness degradation at night and solved the problem of poor texture details of visible images in night scenes [22]. Tang et al. proposed a network structure that combines a segmentation network and a fusion network, named SeAFusion, which cascaded the fusion network and the segmentation network. The segmentation loss is used to guide the fusion module, so that the fused image contains more semantic information, which effectively improves the performance of the fused image in visual tasks [23]. Hao et al. proposed a novel image fusion network named MFTCFNet, which aimed to solve the problems of target edge blurring and feature loss [24]. Hao et al. proposed a fusion method called VDFEFuse, which aimed to solve the problems of energy loss and edge blurring that occur in traditional fusion methods [25].

GAN can learn to generate realistic fused images that simulate real image feature distribution. Ma et al. used GAN to the field of image fusion for the first time and redefined the image fusion problem as an adversarial game between a generator and a discriminator. The generator is mainly to generate a fused image containing infrared intensity and visible gradient, and the discriminator is to force the image generated by the generator to have more texture details [26]. Xu et al. proposed a network with two discriminators designed to distinguish structural differences and content loss between the fused image and the source images. The fused image is motivated to preserve more information [27].

Transformer is a deep learning model used in fields such as natural language processing and computer vision. It was first proposed for machine translation tasks, but has now been widely used in many visual tasks, such as target detection, image segmentation, etc. Due to its strong ability to capture context, Transformer has been widely used in multimodal image fusion and has achieved good fusion performance. Wang et al. proposed a fusion network with the residual swin Transformer, in which the global attention features obtained using the Transformer have a strong representation of infrared targets and visible image detail information [28]. Zhao et al. proposed a correlation-driven feature decomposition fusion network named as CDDFuse, which introduced a dual-branch feature extractor. The CNN branch is used to process high-frequency localized information, and the Transformer branch is used to process low-frequency global features [29].

The fusion of infrared and visible images aims to synthesize the advantages of source images to produce the fused images with target information, rich details, and high contrast. However, the existing algorithms often neglect the synchronization of local and global feature extraction, which results in the loss of critical information in the fused image. To address this problem, a hybrid dual-branch fusion network combining CNN and Transformer is proposed in this paper. First, a local feature extraction module with CNN as the core is constructed, and the residual gradient module is used to enhance the ability of network to extract texture information, and jump connections and coordinate attention are used to associate shallow features with deep features. Second, a global feature extraction module based on Transformer is constructed to capture global contextual information of the image. After the verification of different experimental datasets, the proposed method has better performance than most of the current state-of-the-art fusion algorithms.

## 3. Proposed Method

In this section, we will give a detailed description of the network framework of the proposed method. First, we describe the overall idea of the proposed method. Then, the composition of each subnetwork module is presented. Finally, the loss function used in this paper is described.

### 3.1. *Network Overview*

**Figure 1:**
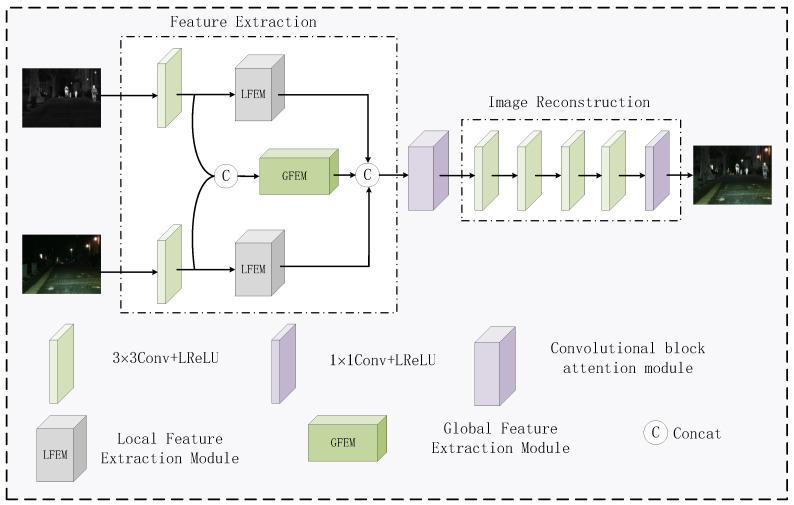
General framework of the proposed network.

The proposed fusion network is illustrated in Figure 1. It mainly consists of feature extraction and image reconstruction. Taking a pair of infrared image $I_{ir}$ and visible image $I_{vi}$ from the same scene as input, and after going through the fusion network, a fused image $I_{f}$ is obtained that contains both infrared target information and visible texture detail information. More specifically, our feature extraction part consists of three parts: a convolutional layer, an LFEM, and a GFEM. The infrared image feature $\phi_{\mathrm{ir}}$, visible image feature $\phi_{\mathrm{vi}}$, and global feature ϕ are obtained after the source images going through the feature extraction module. The splicing strategy is used to fuse these three features to get the hybrid features $\phi_{f}$. Next, these hybrid features are processed through a dual-channel attention module (CBAM) [30], which is capable of focusing on both channel information and spatial information. Finally, the fused image is obtained through the image reconstruction network. The image reconstruction part comprises four consecutive 3 × 3 convolutional layers, succeeded by a 1 × 1 convolutional layer. Within the 3 × 3 convolutional layers, Leaky Rectified Linear Unit (LReLU) activation functions are employed, while a Tanh activation function is utilized for the 1 × 1 convolutional layer. Table 1 shows the specific parameters of some of the network modules.

### 3.2. Model Details

#### 3.2.1. Local Feature Extraction Module

**Figure 2:**
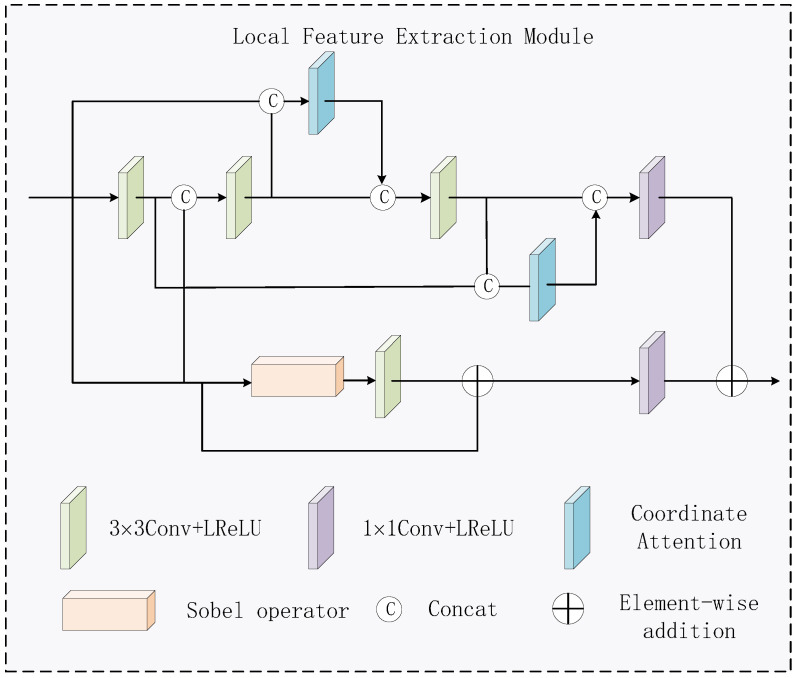
Local feature extraction module.

The network architecture of LFEM is shown in Figure 2. The backbone network consists of four convolutional layers and two coordinate attention modules that use jump links. Among them, the first three convolutional layers use kernel with size of 3 × 3, and the latter one uses kernel with size of 1 × 1. The residual stream is composed of a sobel operator and a 3 × 3 convolutional layer in series before residual linking. Then, the same number of channels as in the backbone network is obtained by a 1 × 1 convolutional layer and finally summed. The activation functions of the convolutional layers are LReLU. The LFEM module is designed to effectively extract the local feature information to make the fused image contain rich detail contents, which aims to avoid the loss of key information. The residual gradient module plays a role in extracting texture features. The jump connections create direct connectivity channels between different layers of the network, allowing shallow features to be passed directly to deeper layers of the network. Coordinate attention enables the network to pay attention to the feature information at different locations and their interrelationships, making the extracted local features more complete.

The residual gradient module consists of the Sobel operator and the 3 × 3 convolution, which is realized by residual concatenation. The Sobel operator, as a discrete differential operator, detects the edges by calculating the rate of grayscale change on each pixel in the horizontal and vertical directions of the image. By combining the horizontal and vertical gradient components, the gradient magnitude and direction can be obtained to highlight the edge information. When the features processed by the Sobel algorithm are then subjected to the 3 × 3 convolution operation, the local image details can be effectively extracted, which greatly enhances the ability to perceive image details. The residual connection can alleviate the problem of gradient vanishing. In addition, it can make the features in the lower layer directly transfer to the higher layer, which significantly improves the feature extraction ability of the network.

**Figure 3:**
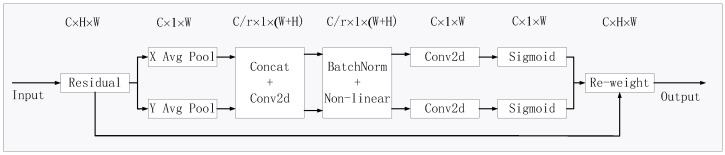
Coordinate attention module.

The architecture of the CA module is shown in Figure 3. The CA module can be viewed as a computational unit which can decompose channel attention into two 1D feature encoding processes. With this approach, long-range dependencies can be captured along one spatial direction, while more precise positional information can be retained along another spatial direction, leading to enhanced feature extraction capabilities. Specifically, for an input *X*, each channel can be encoded separately along the horizontal and vertical coordinates [31]. Thus, the output of the *c*-th channel at height *h* can be formulated as:

(1) $$z_{c}^{h}\left(h\right)=\frac{1}{W}\sum_{0\leq i\leq W}x_{c}\left(h,j\right)$$

Similarly, the output of the *c*-th channel at width *W* can be formulated as:

(2) $$z_{c}^{w}\left(w\right)=\frac{1}{H}\sum_{0\leq j\leq H}x_{c}\left(j,w\right)$$

For the output calculated by the above formula, splicing is performed. Then, it is computed by the convolutional transform function $F_{1}$. The computational process can be formulated as:

(3) $$f=\delta \left(F_{1}\left(\left[z^{k},z^{w}\right]\right)\right)$$

where $\left[\cdot ,\cdot \right]$ denotes a cascade operation along the spatial dimension, δ is a nonlinear activation function, $F_{1}$ is a 1 × 1 convolutional transform function, and $f\in R^{C/r\times (H+W)}$ is an intermediate feature map that encodes spatial information in the horizontal and vertical directions, where *r* is the reduced ratio of the control block size. The intermediate feature map *f* is decomposed along the spatial dimension into two separate tensors $f^{h}\in R^{C/r\times H}$ and $f^{w}\in R^{C/r_{\times }W}$. The tensor $f^{h}$ in the horizontal dimension and the tensor $f^{w}$ in the vertical dimension are transformed into tensors with the same channel as the input *X* by means of convolutional transformation functions $F_{h}$ and $F_{w}$ with kernel 1 × 1. The computational process can be formulated as:

(4) $$g^{h}=\sigma \left(F_{h}\left(f^{h}\right)\right)$$

(5) $$g^{w}=\sigma \left(F_{w}\left(f^{w}\right)\right)$$

where σ is the sigmoid function. The final output of the coordinate attention module CA is $y_{c}\left(i,j\right)$, which can be formulated as:

(6) $$y_{c}\left(i,j\right)=x_{c}\left(i,j\right)\times g_{c}^{h}\left(i\right)\times g_{c}^{w}\left(j\right)$$

#### 3.2.2. Global Feature Extraction Module

**Figure 4:**
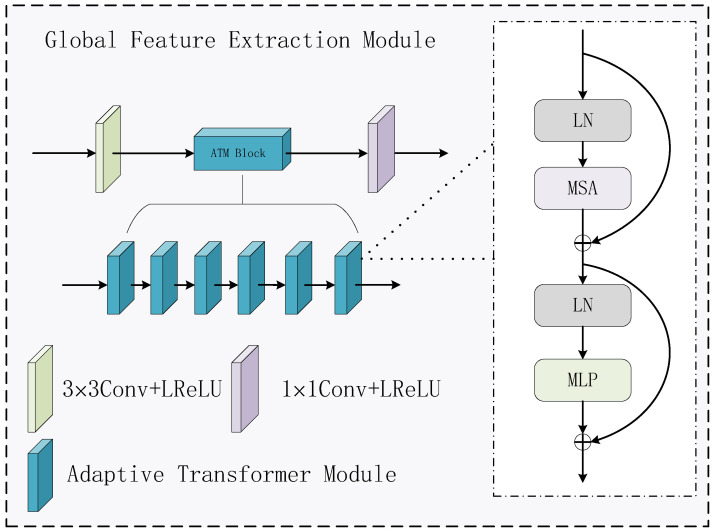
Global feature extraction module.

The network architecture of GFEM is shown in Figure 4. Since the Transformer has a strong ability to extract global information, we use the Adaptive Transformer (ATM) [32] and two convolutional layers to form the GFEM. Specifically, the input features pass through the convolutional layer, the six ATM modules, and the convolutional layer in turn, thus obtaining the deep features that contain global information. The kernel size of the former convolutional layer is 3 × 3 and the latter convolutional layer is 1 × 1. The activation functions are LReLU. The GFEM module is responsible for capturing the global contextual information of the images, fully extracting the features of the images at the overall level, ensuring that the fused image can contain enough information from a macro perspective, and cooperating with the LFEM to make the fused image better in terms of details and overall information presentation. The self-attention mechanism of the Transformer is capable of calculating the degree of correlation between each location and all the other locations of the image.

The architecture of ATM is shown at the right side of Figure 4. ATM consists of two addition operations. The first addition operation can be formulated as:

(7) $$\phi_{ATM1}^{\mathrm{Out}}=MSA\left(LN\left(\phi_{ATM}^{\mathrm{In}}\right)\right)+\phi_{ATM}^{\mathrm{In}}$$

where $\phi_{\mathrm{ATM}1}^{\mathrm{Out}}$ and $\phi_{ATM}^{\mathrm{In}}$ denote the output and input, respectively, LN denotes the normalization, and MSA denotes the multi-head attention. The second addition can be formulated as:

(8) $$\phi_{ATM}^{Out}=MLP\left(LN\left(\phi_{ATM1}^{Out}\right)\right)+\phi_{ATM1}^{Out}$$

where $\phi_{ATM}^{Out}$ denotes the final output and MLP denotes multilayer perception.

### 3.3. Loss Function

In order to ensure that the fusion network has a good fusion performance, the loss function consists of intensity loss and texture loss. [23]. It is defined as follows:

(9) $$L_{C}=L_{I}+\alpha L_{T}$$

where α denotes the hyperparameter that balances between intensity loss and texture loss. Intensity loss is mainly used to ensure that the generated fused image is as similar as possible to the target image in terms of overall brightness and contrast, thereby maintaining the overall visual perception consistency of the image. The texture loss is used to preserve edge information and subtle textures in the fused image, helping the model learn clearer and more accurate image details. Thus, the intensity loss can be defined as:

(10) $$L_{I}=\frac{1}{HW}{∥I_{f}-\max\left(I_{ir},I_{vi}\right)∥}_{1}$$

where *H* and *W* denotes the length and width of the image, respectively, max0 denotes the maximum value of the element to be sought, and ${∥\cdot ∥}_{1}$ denotes the $L_{1}$-norm. For pixels at the same location, we select the pixel with higher intensity value as the corresponding pixel for fusion. In the image fusion task, we hope to obtain a fused image with rich texture details and accurately reflect the target heat distribution. However, the intensity loss function provides only a rough constraint on the overall luminance distribution and does not preserve texture details well. Therefore, we need to introduce a texture loss to induce the fused image to preserve edge information and subtle textures. The texture loss is defined as:

(11) $$L_{T}=\frac{1}{HW}∥\left|\nabla I_{f}\right|-\max\left(\left|\nabla I_{ir}\right|,|\nabla I_{vi}\right|{)∥}_{1}.$$

where ∇ denotes the Sobel operator and |·| denotes absolute value computation. The Sobel operator is an image processing filter that efficiently finds edges and contours in an image by calculating the gradient of each pixel, thus preserving the textural details of the image.

## 4. Experimental

This section first briefly outlines the datasets and experimental methods. Subsequently, a comparative analysis of the proposed method with seven state-of-the-art image fusion algorithms is presented. The fusion results on three datasets are subjectively and objectively evaluated. In addition, a series of ablation experiments are performed to further demonstrate the effectiveness of the proposed method.

### 4.1. Experimental Configurations

We used 1295 pairs of infrared and visible images as the training set, with 1083 pairs from the MSRS dataset [23], 170 pairs from the RoadScene dataset [33], and 42 pairs from the TNO dataset [34]. All these 1295 pairs of images are cropped to a 340 × 256 size. Thirty pairs of images from the MSRS dataset, ten pairs of images from TNO, and ten pairs of images from RoadSence are used as the test images, respectively. In order to assess the effectiveness of the fusion algorithms in this paper, objective and subjective evaluations are performed using the test set described above. Seven state-of-the-art fusion algorithms are used to compare with the proposed method. These seven methods are DeepFuse [35], DenseFuse [18], RFN-nest [19], SeaFusion [23], SwinFuse [28], U2 [36], and ITFuse [37]. The codes of the above methods are publicly available and the parameters are set according to the given parameters in corresponding paper. Eight commonly used indicators for objective image assessment are selected, including information entropy (EN) [38], mutual information (MI) [39], spatial frequency (SF) [40], average gradient (AG) [41], standard deviation (SD) [42], visual information fidelity (VIF) [43], edge information (Qabf) [44], and structural similarity (SSIM) [45]. The higher the value of the indicator, the better the fusion.

Throughout the training process, the size of BatchSize is set to 2, the learning rate is 0.001, Epoch is 15, and the optimizer is Adam. The experiments in this paper are conducted on a computer configured with Windows 10 (64-bit), NVIDIA GeForce RTX 3060 GPU, Intel(R) Core(TM) i7-11700, and 16 GB of RAM.

### 4.2. Experimental Results and Analysis

#### 4.2.1. MSRS

**Figure 5:**
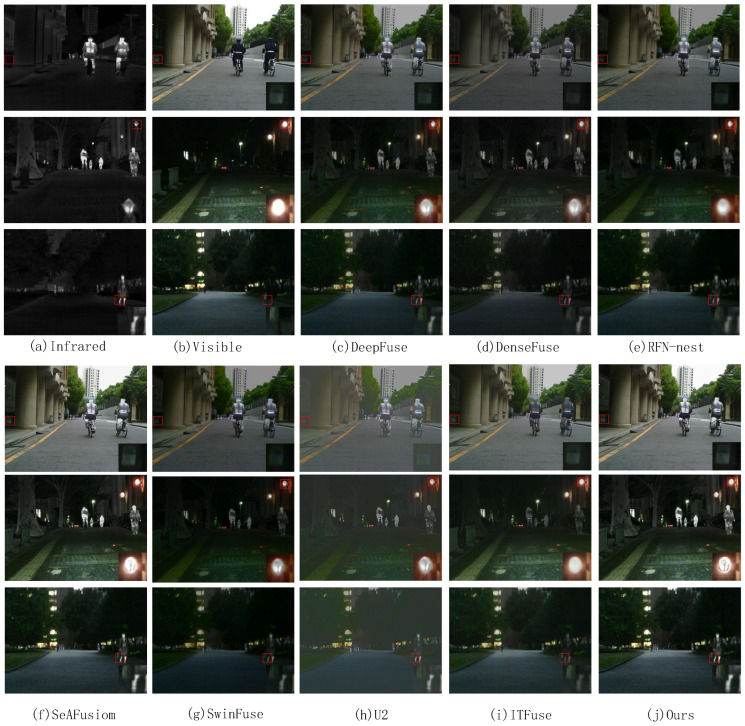
Subjective comparison of three pairs of images on the MSRS dataset. (**a**) Infrared, (**b**) Visible, (**c**) DeepFuse, (**d**) DenseFuse, (**e**) RFN-nest, (**f**) SeaFusion, (**g**) SwinFuse, (**h**) U2, (**i**) ITFuse, (**j**) Ours.

On the MSRS dataset, we select 30 pairs of images from different scenes for testing. We visualize the fusion results of three pairs of images and show the visualization results in Figure 5. The proposed method is compared subjectively with seven state-of-the-art methods on the MSRS data for three pairs of images. We select an area in the fused image and enlarge it to clearly compare the fusion results. The magnified region is marked with a red box, and the magnified image is placed in the lower right corner of the original image. In Figure 5, the fused images obtained by DenseFuse and SwinFuse reflect the texture details in the source images, but the images are dark overall. The fused images from DeepFuse and RFN-nest are clearer, but do not preserve enough information about the texture details. The U2 and ITFuse methods preserve more detail information, but introduce a lot of noise into the images, resulting in an unclear image. Although the images obtained by SeAFusion and ours both retain the target information and texture detail information, the proposed method is clearer in the detail texture.

**Figure 6:**
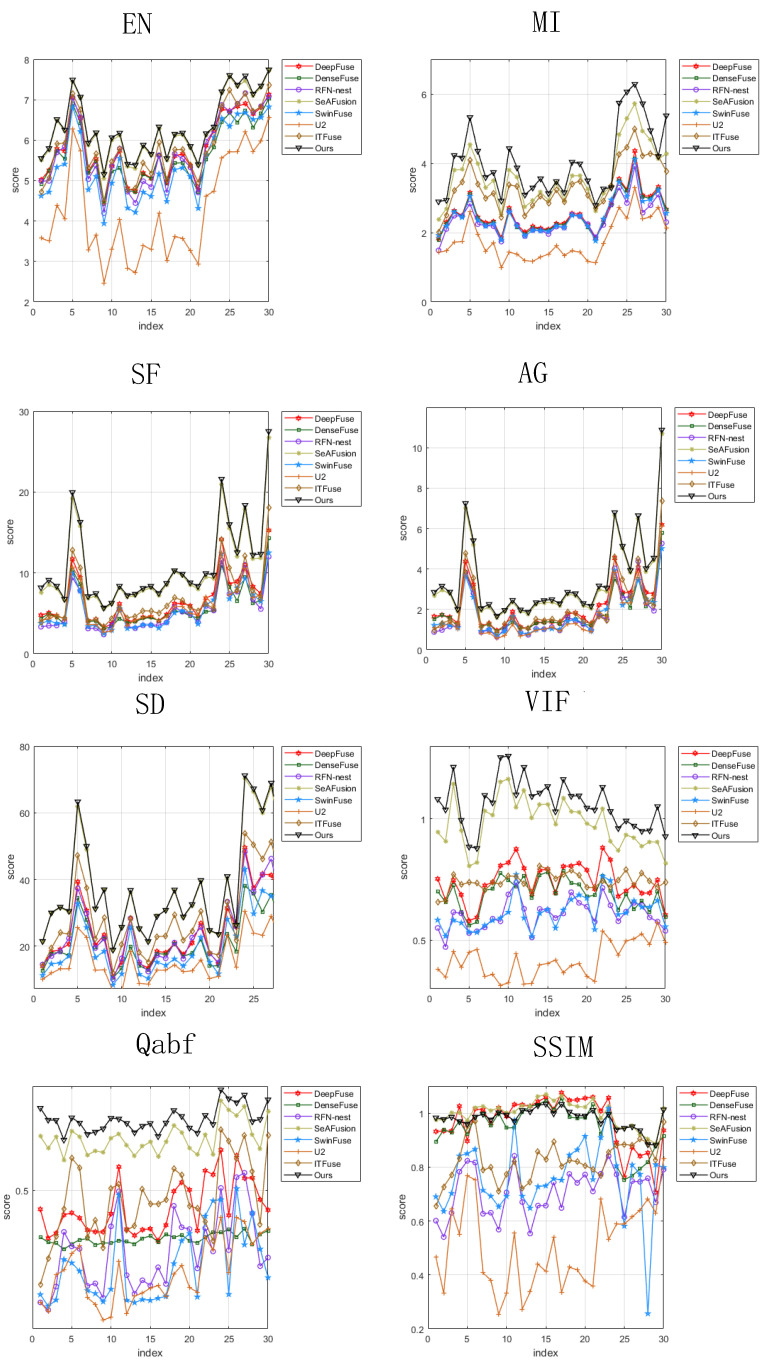
Objective comparison of eight indicators on ten image pairs from the MSRS dataset.

For objective evaluation, eight commonly used image evaluation indicators are used to assess the fusion performance of the seven methods. Figure 6 illustrates the values of the metrics after fusion of 30 pairs of images. It can be seen that most of the metrics of the proposed methods are in the lead. Table 2 gives the average values of each metric for the 30 fused images, where the best value is marked in bold and the second best value is underlined. As can be seen from Table 2, the proposed method achieves the highest value compared to the other seven methods in terms of EN, MI, SF, AG, SD, VIF, and Qabf. For the SSIM indicator, the SeAFusion method achieves the highest value, followed by the proposed method. In summary, the proposed method achieves significant advantages on multiple evaluation metrics and has a good fusion performance on the MSRS dataset.

#### 4.2.2. TNO

**Figure 7:**
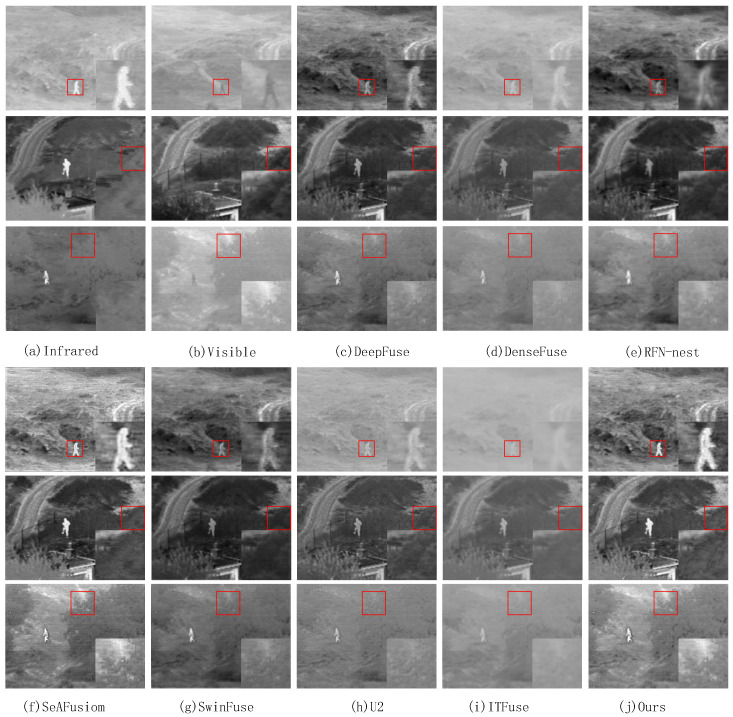
Subjective comparison of three pairs of images on the TNO dataset. (**a**) Infrared, (**b**) Visible, (**c**) DeepFuse, (**d**) DenseFuse, (**e**) RFN-nest, (**f**) SeAFusion, (**g**) SwinFuse, (**h**) U2, (**i**) ITFuse, (**j**) Ours.

On the TNO dataset, we select ten pairs of representative images for testing and show the fusion results of three pairs of images, as shown in Figure 7.

Figure 7 shows the subjective comparison results between the proposed method and the seven state-of-the-art methods on the three pair of TNO images. We select an area in the fused image and enlarge it to clearly compare the fusion results. The region is marked with a red box and the zoomed image is located in the lower right corner of the original image. As shown in Figure 7, the fused images obtained by DenseFuse and U2 introduce a lot of noise information, resulting in an unclear image. The fused images obtained by DeepFuse and SwinFuse are clearer, but the targets are not significant, and the image as a whole is darker. The RFN-nest and SwinFuse methods preserve more detail information, but the target information is not obvious. The texture of the fused images obtained by ITFuse are not clear and the images are dark. The fused images obtained by SeAFusion have clear detail texture, but the preservation of target information needs to be improved. The proposed method retains more important target information and texture detail information.

**Figure 8:**
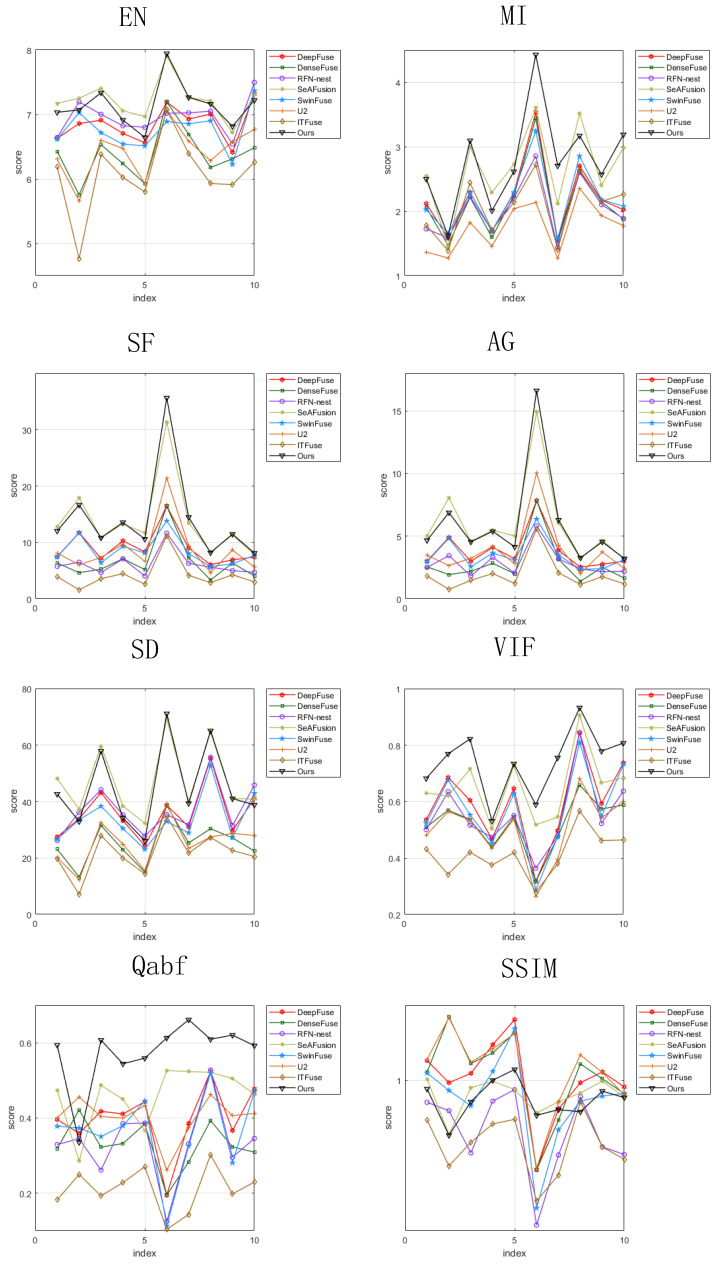
Objective comparison of eight indicators on ten image pairs from the TNO dataset.

For objective evaluation, we use eight image evaluation metrics to assess the fusion performance of these eight methods. Figure 8 shows the values of the metrics for ten pairs of fused images in the TNO dataset. It can be seen that most of the fusion metrics of the proposed method are ahead of that of the other methods. The average values of the metrics for ten fused images are given in Table 3, where the best values are marked in bold and the second best metrics are underlined. Compared with the other seven methods, the proposed method is better than the other methods in terms of MI, SF, VIF, and Qabf. In terms of SSIM, the proposed method is slightly inferior to the other methods. In conclusion, the proposed method are superior in most evaluation indicators, preserves more information, and have good fusion performance on the TNO dataset.

#### 4.2.3. RoadScene

**Figure 9:**
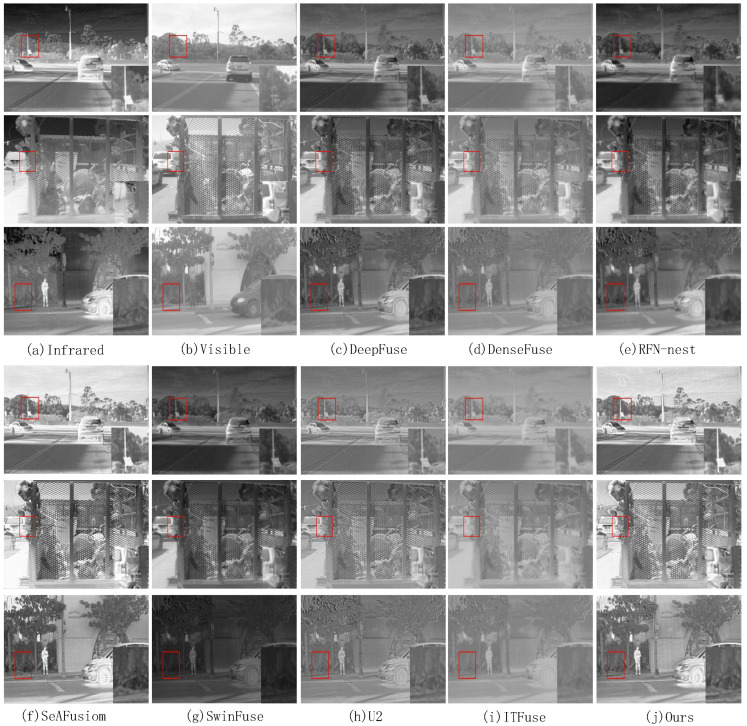
Subjective comparison of 3 pairs of images on the RoadSence dataset. (**a**) Infrared, (**b**) Visible, (**c**) DeepFuse, (**d**) DenseFuse, (**e**) RFN-nest, (**f**) SeAFusion, (**g**) SwinFuse, (**h**) U2, (**i**) ITFuse, (**j**) Ours.

On the RoadScene dataset, we select ten representative image pairs for testing. As shown in Figure 9, we select three groups of fusion results for visualization. Figure 9 demonstrates a subjective comparison between the proposed method and seven state-of-the-art methods on three pairs of images from the RoadSence dataset. We select an area in the fused image and enlarge it to clearly compare the fusion results. The region is marked with a red box, and the zoomed image is located in the lower right corner of the original image. The fused images obtained by the SwinFuse method have good texture details, but the images are dark overall. The fused images obtained by DenseFuse and RFN-nest do not reflect more texture details. The fused images obtained by U2 and DeepFuse have clear texture details. The fused images obtained by ITFuse have an unclear texture. The fused images obtained by SeAfusion have good texture details but the brightness is dark. The fused images obtained by the proposed method are slightly inferior to DeepFuse and SeAfusion in terms of texture details, but have an advantage in terms of overall brightness.

**Figure 10:**
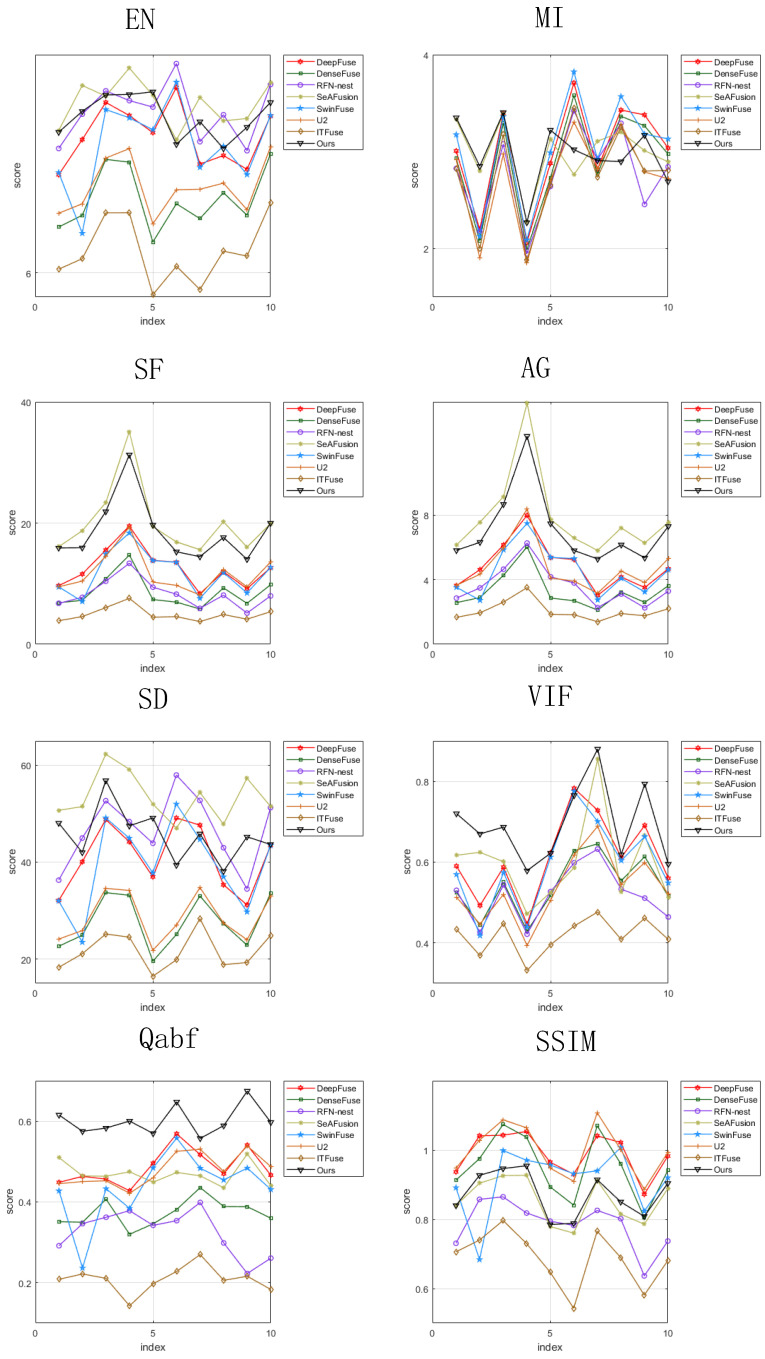
Objective comparison of eight indicators on ten image pairs from the RoadSence dataset.

For objective evaluation, we use eight common image evaluation metrics to assess the fusion performance of these fusion methods. Figure 10 shows the values of the metrics for ten pairs of fused images. Table 4 gives the average values of the metrics for the ten groups of fused images, where the best values are marked in bold and the second best values are underlined. From Table 4, it can be seen that the proposed method is ahead of the other methods in terms of VIF and Qabf. In terms of EN, MI, SD, and SSIM metrics, the proposed method is slightly inferior to the other methods. In conclusion, the proposed method have good performance on RoadScene dataset.

### 4.3. Ablation Experiments

#### 4.3.1. LFEM Module Ablation Experiments

**Figure 11:**
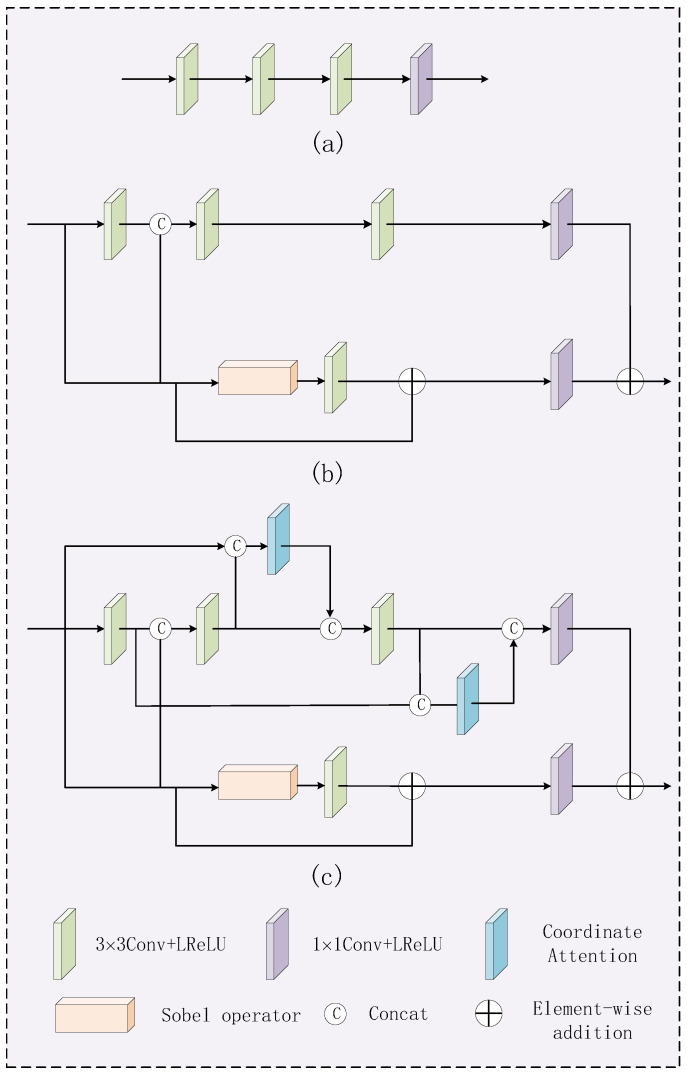
Network framework for LFEM ablation experiments. (**a**) The network only with convolutional layers, (**b**) The network with the residual gradient module, (**c**) The network with the residual gradient module and CA.

In order to verify the role of the residual gradient module and CA in the LFEM module, we conduct ablation experiments on different parts of the LFEM module, which keeps the network architecture of the feature reconstruction part unchanged during the experiments. The first ablation experiment takes the network architecture shown in Figure 11, where Figure 11a represents a backbone network only with convolutional layers, Figure 11b represents the network with the residual gradient module, and Figure 11c represents the network with the residual gradient module and CA.

For objective evaluation, eight commonly used image evaluation metrics are used to assess the fusion performance of different networks. Thirty pairs of images from the MSRS dataset are used for testing. The objective evaluation results are shown in Table 5, where the best values are highlighted in bold and the second best values are underlined. As listed in Table 5, the proposed network shown in Figure 11c has the best values in terms of EN, MI, SF, AG, SD, VIF, and Qabf. It can be concluded that the LFEM module has important role for improving the fusion performance.

#### 4.3.2. Ablation Experiments with the GFEM Module

**Figure 12:**
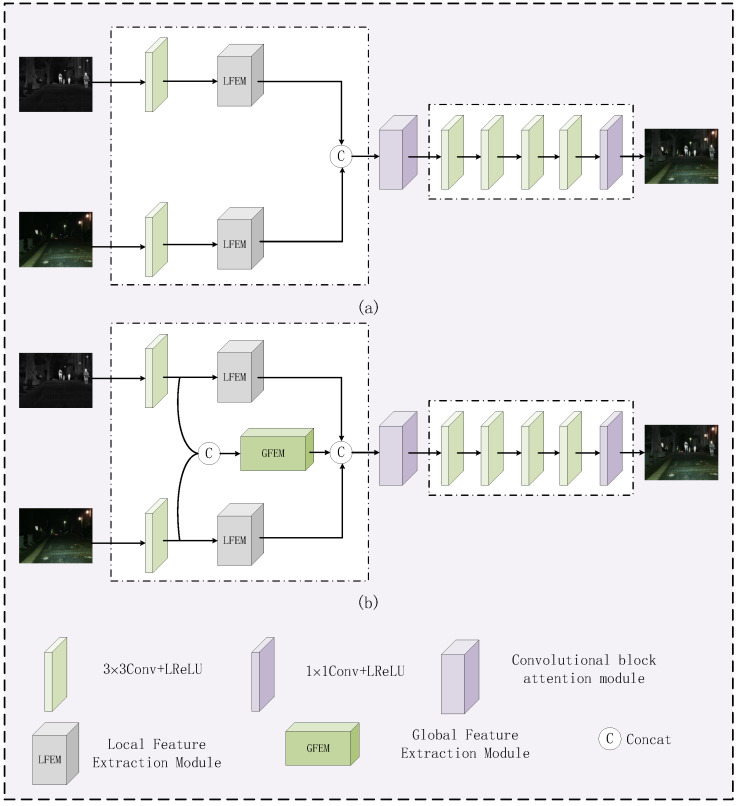
Network framework for the GFEM ablation experiments. (**a**) The proposed network without the GFEM, (**b**) The proposed network.

Ablation experiments are performed to verify the role of the GFEM in the network. The network architecture of the ablation experiment is shown in Figure 12, where Figure 12a indicates that the proposed network without the GFEM and Figure 12b indicates the proposed network. The objective evaluation results are shown in Table 6, where the best values are highlighted in bold. It can be observed that the proposed network with the GFEM provides the best values in terms of EN, SF, AG, SD, VIF, Qabf, and SSIM. It can be concluded that the GFEM is effective for improving the fusion performance.

## 5. Conclusions

In this paper, we proposes a dual-branch fusion method combining CNN and Transformer for infrared and visible images fusion, which design the LFEM and GFEM modules. In the LFEM module, the residual gradient module is used to enhance the representation of the fusion network for gradient texture information, while jump connections and CA are used to enhance the connection between shallow and deep features. In the GFEM module, Transformer is used to capture global contextual information. Experimental results show that the proposed algorithm outperforms the other fusion algorithms in terms of performance. In most of the studies, the quality of the fused images is emphasized while ignoring the needs of advanced visual tasks. In future work, visual tasks can be combined with fusion algorithms to motivate better application of fused images in visual tasks. 

## Figures and Tables

**Table 1:** Specific parameters of the network modules.

Module	Layer	InChannel	Kernel	OutChannel
Conv	Conv	1	3 × 3	16
LFEM	Conv1	16	3 × 3	16
	Conv2	32	3 × 3	16
	Conv3	48	3 × 3	16
	Conv4	48	1 × 1	64
	Conv5	16	3 × 3	16
	Conv6	16	1 × 1	64
	CA1	32	-	32
	CA2	32	-	32
	Sobel	16	-	16
CBAM	CBAM	192	-	192
GFEM	Conv1	32	3 × 3	48
	ATM Block	48	-	48
	Conv2	48	1 × 1	64
Image Reconstruction	Conv1	192	3 × 3	128
	Conv2	128	3 × 3	64
	Conv3	64	3 × 3	32
	Conv4	32	3 × 3	16
	Conv5	16	1 × 1	1

**Table 2:** Average objective evaluation values of different methods on the MSRS dataset.

	EN	MI	SF	AG	SD	VIF	Qabf	SSIM
DenseFuse	5.6318	2.5076	5.7756	1.9104	22.0752	0.6928	0.3598	0.4716
DeepFuse	5.7797	2.5585	6.6139	2.1704	25.5368	0.7416	0.4511	0.4856
RFN-nest	5.7309	2.396	5.4399	1.7774	25.3292	0.6063	0.3243	0.3535
SeaFusion	6.3032	3.6526	10.5822	3.3955	38.9243	0.9828	0.6501	**0.5003**
SwinFuse	5.4255	2.4841	5.5429	1.7685	21.2713	0.6141	0.2813	0.3792
U2	4.2659	1.7707	6.1024	1.7538	16.4494	0.4181	0.2625	0.2525
ITFuse	5.8636	3.3454	6.9037	2.1408	29.6505	0.7416	0.4754	0.4123
Ours	**6.3499**	**4.0457**	**10.9529**	**3.5238**	**39.3671**	**1.0617**	**0.7046**	0.4902

**Table 3:** Average objective evaluation values of different methods on TNO dataset.

	EN	MI	SF	AG	SD	VIF	Qabf	SSIM
DenseFuse	6.3743	2.1185	6.6079	2.8099	24.9774	0.5213	0.3283	0.508
DeepFuse	6.8532	2.1949	9.0818	3.8208	35.7965	0.5933	0.3975	0.5007
RFN-nest	6.9603	2.0321	6.1295	2.8885	36.9812	0.5524	0.3333	0.3942
SeAFusion	**7.2254**	2.6623	13.8844	**6.0214**	**47.1595**	0.6519	0.4603	0.4694
SwinFuse	6.7661	2.1879	8.4439	3.485	33.6938	0.5688	0.3638	0.4614
U2	6.4249	1.7426	8.7519	3.8859	25.2241	0.5038	0.401	**0.518**
ITFuse	6.0729	2.0627	4.1679	1.9084	21.6013	0.4142	0.2102	0.3702
Ours	7.1412	**2.7897**	**14.1548**	5.9568	44.9232	**0.7407**	**0.5738**	0.4608

**Table 4:** Average objective evaluation values of different methods on the RoadScene dataset.

	EN	MI	SF	AG	SD	VIF	Qabf	SSIM
DenseFuse	6.6093	2.8977	8.5632	3.2868	27.5786	0.5422	0.3727	0.4735
DeepFuse	7.1316	2.9916	12.5806	4.8351	4.08806	0.6114	0.4854	0.4943
RFN-nest	7.3302	2.7605	8.3071	3.6122	46.5545	0.5188	0.3255	0.3924
SeAFusion	**7.3976**	2.9906	**20.1357**	**7.8853**	**53.3587**	0.5986	0.4693	0.4269
SwinFuse	7.0559	**3.0371**	11.7654	4.4932	39.4182	0.5906	0.4376	0.4561
U2	6.7116	2.7263	11.7283	4.7155	28.6758	0.4786	0.4787	**0.4987**
ITFuse	6.1772	2.7582	4.9253	2.0659	21.6782	0.4176	0.2085	0.3439
Ours	7.2867	2.9783	18.6001	7.1006	45.572	**0.6931**	**0.6008**	0.4359

**Table 5:** Average values for each metric in the LFEM module ablation experiment.

	EN	MI	SF	AG	SD	VIF	Qabf	SSIM
Figure 11a	6.2414	4.1042	10.5952	3.2398	37.2066	0.9821	0.6412	0.5045
Figure 11b	6.2896	3.7029	10.5539	3.3483	37.8961	0.9871	0.6379	**0.5202**
Figure 11c	**6.3237**	**4.3594**	**10.9001**	**3.4785**	**39.0817**	**1.0492**	**0.6991**	0.4881

**Table 6:** Average values for each metric in the GFEM module ablation experiment.

	EN	MI	SF	AG	SD	VIF	Qabf	SSIM
Without the GFEM	6.3237	**4.3594**	10.9001	3.4785	39.0817	1.0492	0.6991	0.4881
With the GFEM	**6.3499**	4.0457	**10.9529**	**3.5238**	**39.3671**	**1.0617**	**0.7046**	**0.4902**

## Data Availability

Publicly available datasets were analyzed in this study.
